# Piloting a Pragmatic Randomized Controlled Trial on the Effects of Integrated Psychosocial Care in Intensive Care Units (Phase B of the Integrated Psychosocial Care–Pilot Project): Protocol for a Feasibility Study

**DOI:** 10.2196/77490

**Published:** 2026-06-01

**Authors:** Heike Heytens, Simone Korger, Sophie Felicitas Nickel, Wencke Schindler, Melanie Elgner, Kader Aysu Şahin, Gironimo Krieg, Katrin Schürmann, Julianna Gehrig, Lorena Brenner, Marius Binneböse, Matthias Rose, Harald Gündel, Florian Junne, Christian Apfelbacher

**Affiliations:** 1 Institute of Social Medicine and Health Systems Research, Medical Faculty University Hospital Magdeburg, Otto-von-Guericke University Magdeburg, Leipziger Str. 44, Magdeburg, 39120, Germany, 49 391-6724349, 49 391-6724310; 2 Clinic of Psychosomatic Medicine and Psychotherapy, University Hospital Ulm, Ulm, Germany; 3 Institute of Social Medicine and Health Systems Research, Medizinische Fakultät, Otto-von-Guericke University Magdeburg, Magdeburg, Germany; 4 University Clinic for Psychosomatic Medicine and Psychotherapy, University Medicine, Otto-von-Guericke-University Magdeburg, Medical Faculty, Magdeburg, Germany; 5 Medical Clinic - Department of Psychosomatic Medicine of the Charité, Charité - Universitätsmedizin Berlin, Berlin, Germany

**Keywords:** intensive care unit, psychosocial care, feasibility study, randomized controlled trial, implementation, mixed methods, pilot study

## Abstract

**Background:**

Intensive care units (ICUs) present a highly stressful environment for patients, relatives, and health care professionals, often resulting in psychological distress. Despite the well-documented psychosocial burden, there is a lack of low-threshold, integrated support structures for all target groups. Based on the findings of phase A, a complex intervention was developed involving the permanent integration of study therapists into ICU teams.

**Objective:**

This pilot study (phase B of the Integrated Psychosocial Care [IPS]-Pilot project) aims to assess the feasibility of a pragmatic, cluster–randomized controlled trial (pilot RCT) to evaluate the implementation, acceptability, and potential impact of an integrated psychosocial intervention in adult ICU settings.

**Methods:**

A multicenter, cluster-randomized feasibility trial is conducted across 8 ICUs in Germany (Ulm, Berlin, and Magdeburg). The intervention group received continuous support from study therapists embedded within the ICU team. The control group continues with usual psychosocial care (eg, consultation-based). Mixed methods are applied: quantitative surveys at 2 time points (baseline and follow-up) for health care professionals and relatives, and 1-time follow-up for patients; qualitative interviews with study therapists and health care professionals after 4 months. Additional continuous documentation of the intervention process is conducted by study therapists. Data collection was pseudonymized and managed via secure, General Data Protection Regulation–compliant infrastructure.

**Results:**

Funding for the IPS-Pilot project was awarded in January 2023. Recruitment and baseline data collection for phase B began in July 2024 and were ongoing at the time of manuscript submission (May 2025). By March 2025, 177 health care professionals and 82 relatives had completed baseline questionnaires. In addition, all planned qualitative process interviews with all 4 study therapists and 22 health care professionals were conducted in month 4 of the intervention. Data collection took place through June 2025. The initial study results are scheduled to be published in 2026, following the completion of data analysis.

**Conclusions:**

Preliminary findings suggest that the intervention may be feasible and well accepted. The study design enables the identification of contextual barriers and facilitators, informing the development of a future effectiveness trial. Methodological triangulation ensures robust insights into real-world implementation challenges. Final results will contribute to the design of a subsequent definitive trial.

## Introduction

### Background

Intensive care units (ICUs) are characterized by a high level of psychosocial stress, which represents a considerable challenge for patients, relatives, and health care professionals. This burden manifests in anxiety, stress reactions, and other psychological symptoms and can affect both the quality of life of those affected and the quality of medical care. Patients in ICUs often experience anxiety, delirium, and posttraumatic stress. Postintensive care syndrome describes the long-term physical, psychological, and cognitive consequences of an intensive care stay and affects up to 50% of survivors [1,2]. Relatives are also often under considerable strain: concern about the state of health, uncertainty in medical decisions, and emotional overload can lead to symptoms of anxiety disorders, acute stress reactions, or posttraumatic stress disorder [3]. Health care professionals are also severely affected: time pressure, difficult ethical decisions, and constant confrontation with serious illnesses increase the risk of mental illnesses such as burnout, depressive symptoms, and secondary traumatization [4].

During the COVID-19 pandemic, psychosocial support services for health care professionals were temporarily introduced in many hospitals. However, these measures were mostly temporary and not sustainably integrated into everyday hospital life [5,6]. Although the psychosocial burden in ICUs is well documented [7], there is still a lack of standardized and permanently integrated support structures that address all 3 affected groups—patients, relatives, and health care professionals—together [8,9].

### Relation to Phase A

In phase A of the IPS-Pilot project, the psychosocial needs of patients, relatives, and health care professionals in intensive care were systematically surveyed using qualitative interviews, focus groups, and a quantitative survey [10,11]. The focus was not on the direct survey of stress experiences but on the analysis of support needs, as well as hindering and supporting factors for psychosocial care. The following key challenges were identified: (1) lack of continuous psychosocial support for all target groups, (2) insufficient communication and decision support, and (3) high psychological strain on health care professionals without structural relief programs [10].

Based on these results, a complex psychosocial intervention was developed that includes a psychologist as a permanent team member in the ICU. The development was guided by theory (including the conservation of resources theory [12], psychosocial safety climate [13], and the Medical Research Council [MRC] framework [14]) and was carried out in a participatory manner involving all groups involved.

### Necessity of the Feasibility Study

Complex psychosocial interventions in the context of intensive care medicine are associated with numerous practical, organizational, and ethical challenges. Systematic reviews show that existing interventions usually address only 1 target group, while integrated approaches have hardly been systematically investigated to date. At the same time, the implementation of randomized studies in ICUs is difficult—due to the high heterogeneity of patients, ethical concerns, and logistical hurdles, among other things [15-17].

The UK MRC framework for complex interventions, therefore, explicitly recommends an upstream feasibility study before a large-scale effectiveness trial can take place [14]. Phase B of the Integrated Psychosocial Care (IPS)-Pilot project takes up this recommendation and uses a pilot randomized controlled trial (RCT) to test whether the planned intervention can be implemented under realistic conditions and how a subsequent effectiveness design might need to be adapted.

### Objectives

The aim of this pilot study is to test the feasibility of an RCT to evaluate a complex psychosocial intervention in an intensive care context. The study follows the framework for complex interventions outlined by MRC and does not focus on proof of effectiveness, but rather on the practical feasibility of the planned procedures related to both the intervention and pilot RCT.

The objectives include (1) the examination of the recruitability of patients, relatives, and health care professionals; (2) the analysis of the acceptance and perceived usefulness of the intervention; (3) the evaluation of the integration of the intervention into existing clinical processes; (4) the documentation of adherence to the intervention; and (5) the identification of barriers and conditions for success for a subsequent main study.

### Research Questions

The primary research question is “Is a pragmatic RCT to evaluate the IPS-ICU intervention feasible in terms of recruitment, randomization, implementation, and follow-up?”

The secondary research questions are as follows:

- Is the intervention practicable and realizable in the daily clinical routine of an ICU?
- How is the intervention perceived by patients, relatives, and health care professionals?
- What structural, organizational, and logistical challenges are involved in implementation?
- To what extent can the intervention be integrated into existing workflows and team structures in the ICU?
- Is there initial evidence that the intervention has positive effects on the psychosocial safety climate and the well-being of those involved?

## Methods

### Study Design

The reporting of the methodological procedures follows the CONSORT (Consolidated Standards of Reporting Trials) extension for feasibility studies (Checklist 1) [18].

This multicenter, cluster-randomized, controlled feasibility study is being conducted in 8 general ICUs for adults at 3 sites in Germany (Ulm, Magdeburg, and Berlin). The 8 participating wards were determined in advance and selected according to practical criteria, such as minimum capacity, logistical feasibility, and approval from hospital management. Allocation to the intervention or control group was carried out in a 1:1 ratio at the cluster level (ICU) using computer-assisted randomization via the Randomizer tool (Medical University of Graz) [19]. Randomization was performed by the central study coordinator at Ulm University Hospital. Allocation was concealed at the cluster level through the use of a centralized, computer-based randomization procedure. As randomization was conducted at the ICU level prior to participant inclusion, allocation concealment at the individual participant level was not applicable. Both groups receive psychosocial care—either through the established counseling services or via the implemented study therapist.

### Blinding and Risk of Contamination

Patients, relatives, and health care professional participants are not explicitly informed about ward allocation in the study information materials. It was not specified whether the respondents were on an intervention or control ward. Health care professionals are not actively informed about the group assignment either. However, as a psychologist is permanently integrated into the team on intervention wards, group assignment can still be inferred based on the care structure. The project management, ward management, and the study therapist were informed about the group assignment; further communication of the assignment was avoided. Full blinding of health care professionals to group allocation is not feasible in this trial because the permanent integration of a study therapist on intervention wards is visible in daily practice. By contrast, patients and relatives are not explicitly informed about the intervention content or their group assignment. In the informed consent materials, all participating wards are described as providing psychosocial care, and the study is presented as an investigation of emotional health during and after intensive care. Because most patients receive treatment on only one ICU ward and transfers between participating ICUs are rare, they have no direct comparison standard and are therefore unlikely to distinguish intervention from usual care conditions.

To minimize contamination at the organizational level, study therapists are exclusively assigned to intervention wards and do not provide structured psychosocial counseling on control wards. Existing consultation-liaison services on control wards continue unchanged, without additional involvement from the IPS-Pilot team. Any observations deviating from our expectations will be documented via the process documentation.

### Intervention

In the intervention units, study therapists are permanently integrated into the treatment teams with a half-time position. They provide psychosocial support to patients, relatives, and health care professionals and offer various intervention components adapted to individual needs. These components, along with the role and stance of the study therapists, are specified in the IPS intervention manual.

Before the study onset, the study therapists underwent standardized training that included medical fundamentals, typical stressors in ICUs, and the practical application of the intervention manual. Quality assurance is supported through regular external supervision and peer intervision, which alternated weekly.

### Comparison Condition

In the control group, psychosocial care continues to be provided by existing psychosocial services (eg, the consultation service), but without the permanent integration of a psychologist into the team.

### Pilot Effectiveness Evaluation

#### Overview

Although the intervention and its evaluation are closely linked, they represent 2 methodologically distinct components of the project. The intervention involves the permanent integration of clinical study therapists into the intensive care team.

The accompanying study systematically evaluates the feasibility of conducting an RCT to evaluate the intervention. Key criteria for the feasibility of the RCT include participation and dropout rates, data completeness, and the overall practicability of the administered questionnaire. The feasibility of the intervention is assessed in terms of participation, dropout rates, fidelity, and acceptance. Both quantitative and qualitative methods are applied.

The study is aimed at 3 groups in intensive care: patients, their relatives, and health care professionals.

Planned sample sizes are (1) up to 125 patients and their relatives during the course of the study, and (2) approximately 250 health care professionals. The planned sample size is based on feasibility considerations rather than a formal statistical power calculation. In line with recommendations for pilot and feasibility trials, the sample is intended to provide sufficient information on recruitment processes, data completeness, and variability of candidate outcome measures to inform the design of a subsequent definitive trial.

The standardized survey is conducted with health care professionals at 2 measurement points: a baseline survey (*t*_0_), which is administered within 6 weeks of the start of the intervention for health care professionals and a second survey (*t*_1_) at the end of the intervention phase. Relatives and patients are recruited during the patient’s ICU stay; however, only for relatives, a baseline survey (*t*_0_) is administered during that time to avoid placing a high burden on patients. The follow-up survey (*t*_1_) is conducted with relatives and patients 4 months after discharge from the ICU.

#### Inclusion and Exclusion Criteria

General ICUs for adults that had a defined minimum number of treatment places and whose hospital management had expressly agreed to participate in the study were included. Only adult individuals (≥18 y) with the capacity to provide informed consent were eligible to participate in the survey. However, specialized ICUs that already had firmly established integrated psychosocial care structures and wards where other psychosocial intervention studies were being conducted in parallel were excluded.

Participation in the survey is voluntary and takes place regardless of whether the intervention has been used individually. The aim is to systematically assess the potential effects of the intervention from the perspective of all groups involved, using scales that could be used in a future RCT.

#### Measures

The data collected will be used to assess which dimensions could be suitable as primary or secondary end points for a subsequent effectiveness study—for example, subjective well-being, perceived stress, or aspects of the team climate.

At present, no core outcome set exists for psychosocial health in intensive care settings, and there is no consensus on how changes in psychosocial burden among patients, relatives, or health care professionals should be assessed. For this reason, a broad set of theoretically grounded candidate outcomes is required to capture the multidimensional nature of psychosocial strain and recovery in this context.

The selection of multiple psychosocial outcomes reflects the complex and multilevel nature of psychosocial burden in the ICU context, which affects individual well-being, symptoms of anxiety and depression, perceived stress, and team processes among health care professionals. Combining generic well-being and health-related quality of life measures (World Health Organization–Five Well-Being Index [WHO-5] and 12-Item Short Form Health Survey [SF-12]) with brief symptom scales (Patient Health Questionnaire–4 [PHQ-4] and Perceived Stress Scale–10 [PSS-10]) and work-related constructs (psychosocial safety climate, team cohesion, and irritation) allows us to capture different, theoretically grounded dimensions that may respond differentially to the intervention [12,13,20-28]. In line with recommendations for feasibility studies of complex interventions [14,18], this broad set of candidate outcomes will be used to evaluate acceptability, completeness, and distributional properties, and to inform the selection of a smaller number of primary and key secondary endpoints for a future definitive trial. The aim was an economical but, at the same time, differentiated survey of psychosocial stress and well-being dimensions, which could also be considered relevant target variables for a later main study. Given the feasibility focus of this pilot trial, all analyses will be primarily descriptive. Feasibility outcomes (eg, participation rates, drop-out rates, and data completeness) will be summarized using absolute and relative frequencies, as well as means with SDs or medians with IQRs, as appropriate. No formal hypothesis testing or confirmatory effectiveness analyses are planned. Exploratory analyses of candidate outcome measures will be conducted to assess distributional properties and missingness, with the aim of informing endpoint selection for a subsequent definitive trial.

The following questionnaires are used for health care professionals: WHO-5 [20], Psychosocial Safety Climate Questionnaire [21], Questionnaire for Self-Efficacy, Optimism, and Pessimism [22], SF-12 [23], PSS-10 [24], PHQ-4 [25], Erlangen Team Cohesion at Work Scale (validated German version) [16], Irritation Scale for work-related stress consequences [27], and Intention to Leave (items from the NEXT-study) [29]. For patients and relatives, the following questionnaires are used: WHO-5 [20], SF-12 [23], PSS-10 [24], and PHQ-4 [25].

Sociodemographic data such as age, gender, education, professional experience (health care professionals), stay in the ICU (patients and relatives), psychological stress in ICUs, and use of psychosocial support services are collected. In addition to the standardized survey instruments (eg, WHO-5, SF-12, PHQ-4, and PSS-10) and sociodemographic questions, additional questions developed specifically for the target group are collected. These include evaluative assessments of the comprehensibility, relevance, and reasonableness of the individual parts of the questionnaire, as well as attitudes toward psychosocial care in ICUs (eg, the involvement of a ward psychologist or the use of support services).

The additional questions are worded specifically for the target group (health care professionals vs relatives) and embedded within the respective questionnaire structure. A standardized scale was used particularly for the evaluation questions (Likert scales); open text fields are also provided. These items were developed within the project and do not appear as an independent questionnaire, but rather supplement the standardized instruments in a context-sensitive manner.

### Feasibility Evaluation

A key aim of this pilot study is to assess whether psychosocial outcomes can be reliably and meaningfully recorded under RCT conditions, alongside the technical feasibility of data collection, recruitment and dropout rates, and data completeness, and whether the IPS intervention is feasible in terms of participation, dropout, intervention fidelity, and acceptance. In line with methodological guidance on pilot studies and progression criteria for complex interventions [14,18], the predefined thresholds are therefore not treated as rigid statistical decision rules but as pragmatic benchmarks to judge whether a full-scale RCT appears feasible and justifiable.

For the subsequent definitive trial, we will apply a structured “traffic light” progression framework:

- Proceed: if participation rates in all stakeholder groups are 80% or more, dropout is 35% or less, and missing data on core outcome variables are 20% or less, we will consider the trial design and intervention feasible without major modifications.
- Proceed with modifications: if participation rates are between 60% and 79% and/or missing data are between 21% and 30%, we will identify and address modifiable barriers (eg, recruitment processes and questionnaire length) before progressing to a full RCT.
- Do not proceed as planned: if participation rates fall below 60% in key groups or if missing data on core outcomes exceed 30%, a definitive RCT in its current form will not be recommended; instead, additional developmental work or alternative evaluation designs will be considered.

This framework allows us to combine quantitative feasibility indicators with qualitative insights from the process evaluation, enabling us to reach a transparent and evidence-informed decision on whether and how to progress to a subsequent effectiveness trial.

### Qualitative Evaluation of Study Participation

As part of the qualitative accompanying research, additional data are collected on how patients, relatives, and health care professionals perceive the approach to participate in the study, especially in the context of the particularly stressful situation in an ICU. These assessments are derived from the qualitative process evaluation and informal feedback, complementing the evaluation of the practicability and ethical acceptance of the survey instruments, and do not constitute a separate qualitative study component.

### Feasibility of an RCT

#### Overview

The feasibility of the planned study procedures under real clinical conditions is assessed. The assessment of feasibility focuses on 4 core criteria: participation rate, dropout rate, practicability of the survey, and data completeness, as defined in the study registration. The predefined thresholds for participation rates, dropout rates, and data completeness were informed by previous feasibility and pilot trials of psychosocial and organizational interventions in intensive care and acute care settings, which typically report participation rates between 70% and 90% and dropout rates around 20% to 30% across follow-up assessments [30-37].

#### Target Participation Rates

We aim for high participation rates in all interest groups. Specifically, at least 85% of health care professionals [30,31], and 85% of relatives should participate in the baseline survey (*t*_0_), and at least 80% of patients should participate in the follow-up survey (*t*_1_) [31-36]. These rates relate to the planned recruitment numbers (approximately 250 health care professionals and 125 patients and relatives). Participation will be measured at 2 time points: at the beginning of the study (t0) for health care professionals and relatives, and at a later time point (*t*_1_) for all groups. Participation is documented to compare the planned with the actual number of participants.

#### Definition of Participation Rate

For health care professionals and relatives, participation refers to their involvement in the first survey assessment (*t*_0_). Completion of the follow-up survey (*t*_1_) for health care professionals and relatives is evaluated separately as part of the dropout analysis. For patients, participation refers to the completion of the single planned survey (*t*_1_), as patients are surveyed only once and no longitudinal follow-up is foreseen.

#### Dropout Rate

We define dropout as incomplete participation between *t*_0_ and *t*_1_ among those who had originally agreed to participate. For health care professionals and relatives, dropout is assessed across the 2 survey time points; for patients, it refers to the ratio of respondents in the survey (*t*_1_) to the number of recruited patients who provided informed consent during their ICU stay. The dropout rate should not exceed 35% of those who originally consented [30,34,36].

#### Practicability of the Survey

Practicability is assessed using short evaluative items embedded in the t1 questionnaires for all groups. These items capture tolerability (perceived burden and length of the questionnaire), relevance (perceived meaningfulness and usefulness of the questions), and comprehensibility (clarity and understandability of the items).

These indicators allow us to judge whether the survey instruments are acceptable and feasible under routine ICU conditions, and whether adaptations are required for a subsequent definitive RCT.

#### Data Completeness

Another important criterion is the completeness of data collection. For each target group and survey time point, the proportion of missing data will be monitored and reported. Acceptable thresholds are 20% or less missing data for core outcome variables [38].

### Feasibility of the Intervention

The psychosocial intervention used as part of the study was developed to address challenges in intensive care identified in phase A. To evaluate the feasibility of implementing the intervention, we assessed the following 4 core criteria, in alignment with the OSF preregistration [39].

#### Participation Rate

The IPS intervention is conceptualized as a systemic intervention: its implementation is tied to the participating ICU ward rather than individual use. All patients, relatives, and health care professionals on the intervention wards are considered part of the intervention group, regardless of whether they actively use individual intervention components. Both direct participation (eg, use of specific tools) and indirect effects (eg, reduced burden through the psychologist’s presence) are relevant.

Therefore, individual-level participation rates cannot be meaningfully predefined or assessed. Instead, individual participation is evaluated descriptively based on the intervention’s reach and documented activities, such as the frequency and type of contacts recorded by the study therapists.

At the ward level, participation is defined as the successful implementation of the IPS model in the assigned ICU. All ICUs randomized to the intervention arm are expected to implement the intervention as planned, corresponding to a ward-level participation rate of 100%.

#### Dropout Rate

As the IPS intervention is systematically implemented at the ward level and does not include an obligatory number of components to be administered due to its needs-based approach, individual dropout is not defined in the conventional sense. While it is possible to document whether patients, relatives, or health care professionals discontinue contact with the study therapists, this information primarily serves to describe intervention reach rather than to calculate a formal dropout rate. Instead, contact frequency and reasons for noncontinuation are documented descriptively by the study therapists to inform internal evaluation and interpretation of implementation dynamics. Dropout was assessed at the ward level (ratio of intervention wards on which the intervention was implemented but then discontinued). No predefined threshold for dropout was established for the intervention.

#### Fidelity

Fidelity refers to the extent to which the intervention was implemented as intended. This includes adherence to the intervention manual, the documented presence of study therapists according to duty rosters, the correct implementation of intervention modules (eg, delirium prevention and grief counseling), and weekly self-assessments by study therapists regarding their team integration. All procedures are documented centrally and monitored for deviations. A fidelity rate of 80% or more across these components is targeted [31,36,37].

#### Acceptance

The acceptance of the intervention is assessed via participant evaluations collected through standardized questionnaires at the end of the intervention phase. Participants (patients, relatives, and health care professionals) are asked to assess the relevance, support quality, and helpfulness of the intervention. At least 70% of respondents should rate the intervention as “rater helpful” or “very helpful” [32,37].

In addition to the questionnaire-based evaluation at *t*_1_, acceptability among health care professionals was assessed using the Acceptability of Intervention Measure in a separate, anonymous paper-based format. The Acceptability of Intervention Measure was distributed during scheduled team meetings on the intervention wards, with questionnaires completed either on site or returned later by health care professionals who were not present at the meeting. This procedure ensured broad coverage of health care professionals’ perspectives while maintaining anonymity and methodological consistency with the preregistered process evaluation [40].

### Additional Implementation Indicators (For Internal Evaluation)

In addition to the 4 preregistered feasibility criteria, further indicators are used for internal process evaluation:

- Training fidelity: participation of study therapists in initial training, supervision, and intervision.
- Manual adherence: use of basic and toolbox elements as documented; 80% or more correspondence with the manual [37].
- Personnel stability: documentation of staffing changes or early terminations in study therapist roles.
- Completeness of documentation: proportion of missing data in study therapist protocols (threshold ≤20%).
- Perceived barriers to implementation: structural or organizational obstacles recorded by study therapists.
- Additional support needs: documentation of ad hoc psychosocial support (eg, night or weekend).
- Structural classification: contextual ward data (eg, staffing ratio and occupancy).

### Process Evaluation and Methodological Triangulation

To complement the predefined feasibility criteria, we conducted a qualitative process evaluation during the intervention phase. In addition to qualitative interviews, the process evaluation included documentation data from the study therapist, informal field observations by the research team, and contextual notes. Starting from the fourth month after implementation began, guided interviews were conducted with all 4 study therapists and 2 to 3 health care professionals per participating ward. The aim was to explore the acceptance and implementation of the intervention, as well as the pilot-study, in routine clinical settings and to identify contextual influencing factors. This substudy does not constitute an independent feasibility assessment but serves to contextualize and deepen the interpretation of the quantitative results.

### Methodological Triangulation

To gain a comprehensive understanding of feasibility, acceptance, and implementation, we triangulate multiple data sources: standardized questionnaires, qualitative interviews, and continuous process documentation. The details of the data sources are as follows:

- Standardized surveys provide quantitative data on psychosocial well-being, stress, and acceptance.
- Qualitative interviews capture subjective perspectives and contextual factors.
- Continuous process documentation reflects practical aspects, such as implementation challenges, the use of specific intervention modules, flexibility in application, and perceived implementation barriers.

**Figure 1.**
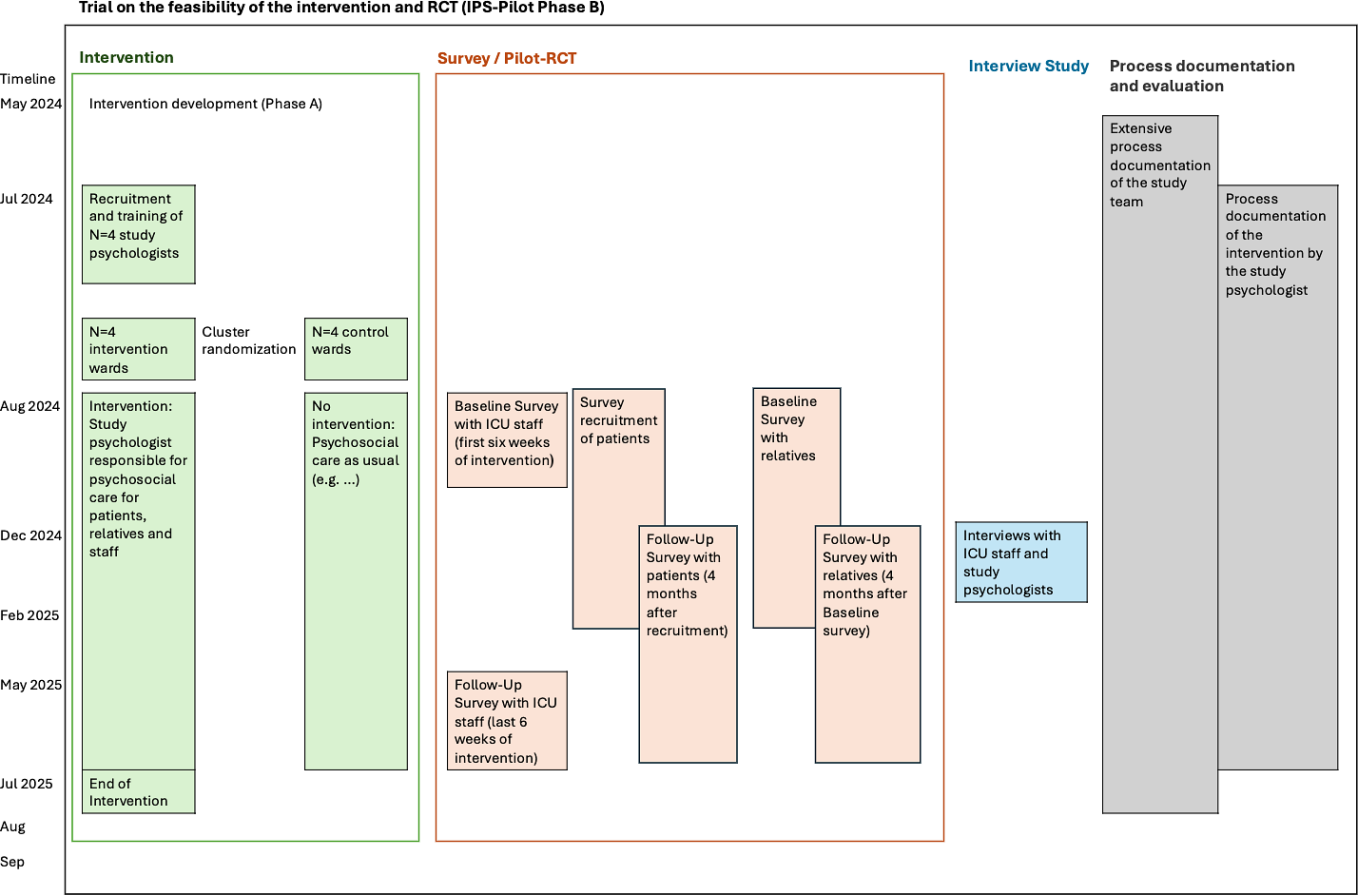
Components and timeline of the study in phase B. ICU: intensive care unit; IPS: Integrated Psychosocial Care; RCT: randomized controlled trial.

Figure 1 illustrates the chronological alignment of data collection with participant groups and methodological components.

Additionally, documentation by STs systematically captures the use of toolbox elements and barriers to implementation. The process data are recorded continuously throughout the intervention phase and are used to support internal quality monitoring.

The triangulation of these sources supports a nuanced interpretation of findings, facilitates the identification of tensions or contradictions, and enhances contextual insight into structural and individual factors influencing feasibility. This integrated perspective provides a foundation for assessing prerequisites for a subsequent effectiveness study.

### Ethical Considerations

Before the start of the intervention, ethical approval for phase B was obtained from the ethics committees of the University of Ulm (112/24) and the University Medical Center Magdeburg. Charité—Universitätsmedizin Berlin accepted these approvals in accordance with §15 (2) of the professional code of the Berlin Medical Association. The study is conducted in accordance with the Declaration of Helsinki and the DFG’s (Deutsche Forschungsgemeinschaft) guidelines for safeguarding good scientific practice, and complies with the European Union General Data Protection Regulation (GDPR) and the Federal Data Protection Act. A comprehensive data protection concept was developed for phase B and approved by the data protection officers at all participating sites. It considers the specific requirements of data collection, storage, and analysis within a multicenter feasibility study. Given the noninvasive nature of the intervention, no serious intervention-related adverse events are expected. Nevertheless, potential psychological burden associated with study participation (eg, distress during questionnaire completion) will be monitored descriptively through participant feedback and process documentation. Any unintended effects will be documented and considered in the overall feasibility assessment.

All personal data collected during the study (eg, interview data) are subject to a 2-stage pseudonymization process. The first pseudonymization takes place locally at the point of data collection. The allocation keys are then managed by an independent trusted third party located at the University Medical Center Magdeburg, which is responsible for storing identifying data separately from study data, organizing follow-up contacts, and ensuring compliance with all data protection requirements.

Reidentification is only possible via the trusted third party. Pseudonymized data are stored centrally, and access to study-relevant data is restricted to authorized scientific staff. A password-protected, access-restricted project platform serves as the technical infrastructure. Upon completion of the study and the expiration of legally mandated retention periods, all data will be deleted in accordance with data protection requirements.

Participation in the study is voluntary and based on written informed consent. Participants are informed of their rights under Articles 13, 14, 15, 17, and 21 of the GDPR, including the right to access their personal data, the right to correct inaccurate data, the right to deletion after study completion, request deletion after study completion, and the right to withdraw consent at any time without providing reasons. No compensation is provided for participation.

### Registration

The study protocol for phase B is registered on the OSF Registries [41] and ClinicalTrials.gov (NCT06733493).

This protocol follows the SPIRIT (Standard Protocol Items: Recommendations for Interventional Trials) 2013 statement (Checklist 2) [42].

- Publications in peer-reviewed, open-access journals, including *JMIR Research Protocols* [10], where the protocol for phase A of the IPS-Pilot project has already been published.
- Presentations at relevant national and international scientific conferences, particularly in the fields of intensive care medicine, psychosomatics, and implementation research.
- Summary reports and targeted dissemination materials for clinical stakeholders and health policy representatives, aimed at informing implementation strategies for integrated psychosocial interventions in intensive care settings.

## Results

The intervention was launched as planned in July 2024 and is being implemented over a period of 12 months in 8 general ICUs. Data collection is staggered and target group–specific as follows:

- Health care professionals: the baseline survey (*t*_0_) was completed in the first 6 weeks of the intervention. A total of 177 participants completed the baseline questionnaire. The follow-up survey (*t*_1_) is scheduled for the end of the intervention period.
- Relatives: The baseline survey (*t*_0_) is completed during the patient’s ICU stay. A follow-up survey (*t*_1_) is conducted 4 months after *t*_0_ to capture delayed psychosocial burden and changes over time. Recruitment for *t*_0_ took place continuously over 8 months and has been completed. A total of 82 fully completed *t*_0_ questionnaires are available. Eleven *t*_1_ responses have been received to date.
- Patients: the survey is conducted once (*t*_1_), 4 months after consent and inpatient stay. In May 2025, 72 consents to participate have been obtained and 6 questionnaires have already been completed and returned.

Parallel to the quantitative data collection, the qualitative accompanying research was conducted during the fourth month of the intervention phase. All planned interviews—with the study therapists and a total of 22 health care professionals from the participating wards—were completed and are currently being analyzed. Discussions were also held with patients and relatives on their perception of the study recruitment. In addition, data were collected on which toolbox measures were actually used and which barriers prevented their use. The weekly study therapist documentation on intervention implementation is ongoing. These process indicators demonstrate that recruitment and intervention delivery are feasible within the planned timeline and will provide the empirical basis for assessing the predefined feasibility criteria.

Data collection took place through June 2025. The initial study results are scheduled to be published in 2026, following the completion of data analysis.

## Discussion

The IPS pilot study investigates a participatory, integrated psychosocial intervention in the intensive care setting that is aimed at patients, relatives, and health care professionals alike. The particularly stressful situation in ICUs has been documented by numerous studies and includes exceptional emotional situations, unclear communication, and high professional demands. Despite this evidence, there is still a lack of structured psychosocial support programs that are integrated into everyday clinical practice on a permanent, interdisciplinary, and low-threshold basis.

This feasibility study examines whether such an intervention can be implemented under real clinical conditions. The study further provides key insights into the feasibility of a randomized study design in an intensive care setting, the acceptance of the intervention among various target groups and its integration into existing workflows. It also identifies the challenges that arise in everyday life, for example, with regard to the availability of psychologists, role clarity, or interaction with existing psychosocial services.

A central methodological goal is to reflect on how to deal with typical limitations of RCTs in complex care situations. These include, for example, limited blinding options, ethical challenges in randomization, and the need to systematically incorporate qualitative feedback. The findings of the feasibility study will contribute to the methodologically sound planning of future efficacy studies and the design of realistic implementation pathways for psychosocial interventions.

It is particularly noteworthy that the intervention is being implemented at 3 university hospitals with different starting points, including 1 location with no previous experience in the field of psychosocial care in the ICU. This multiperspective approach enables an initial assessment of transferability and provides indications of systemic conditions that favor or hinder continuation.

## Supplementary material

10.2196/77490 Checklist 1 CONSORT checklist.

10.2196/77490 Checklist 2 SPIRIT checklist.

## Abbreviations

CONSORT: Consolidated Standards of Reporting Trials
DFG: Deutsche Forschungsgemeinschaft
GDPR: General Data Protection Regulation
ICU: intensive care unit
IPS: Integrated Psychosocial Care (German: Integrierte Psychosoziale Versorgung)
MRC: Medical Research Council
PHQ-4: Patient Health Questionnaire–4
PSS-10: Perceived Stress Scale–10
RCT: randomized controlled trial
SF-12: 12-Item Short Form Health Survey
SPIRIT: Standard Protocol Items: Recommendations for Interventional Trials
WHO-5: World Health Organization–Five Well-Being Index
