# Supplementary material for: Piloting a Pragmatic Randomized Controlled Trial on the Effects of Integrated Psychosocial Care in Intensive Care Units (Phase B of the Integrated Psychosocial Care–Pilot Project): Protocol for a Feasibility Study
Source: JMIR Res Protoc. 2026 Jun 1;15:e77490. doi: 10.2196/77490 (PMC13225221; doi:10.2196/77490)
Supplement: Checklist 1 [file resprot-v15-e77490-s001.docx]

**CONSORT Pilot/Feasibility Checklist**

| Item | Description | Section | Status |
| --- | --- | --- | --- |
| 1a | Pilot trial identification | Title | reported |
| 2a | Background | Introduction | reported |
| 3a | Trial design | Methods | reported |
| 5 | Interventions | Methods | reported |
| 6a | Objectives | Objectives | reported |
| 6b | Progression criteria | Traffic light framework | reported |
| 7a | Sample size rationale | Methods | reported |
| 9 | Allocation concealment | Study Design | reported |
| 12 | Statistical methods | Methods | reported |
| 13a | Participant flow | Results | reported |
| 19 | Harms | Ethics section | reported |
| 20 | Limitations | Discussion | reported |
| 23 | Registration | Registration | reported |
| 25 | Funding | Funding | reported |
