# Supplementary material for: Piloting a Pragmatic Randomized Controlled Trial on the Effects of Integrated Psychosocial Care in Intensive Care Units (Phase B of the Integrated Psychosocial Care–Pilot Project): Protocol for a Feasibility Study
Source: JMIR Res Protoc. 2026 Jun 1;15:e77490. doi: 10.2196/77490 (PMC13225221; doi:10.2196/77490)
Supplement: Checklist 2 [file resprot-v15-e77490-s002.docx]

**SPIRIT Checklist**

| Item | Description | Section | Status |
| --- | --- | --- | --- |
| 1 | Title | Title | reported |
| 2a | Trial registration | Abstract; Registration | reported |
| 3 | Protocol version | Title page | reported |
| 4 | Funding | Acknowledgments | reported |
| 5a | Roles/responsibilities | Authors’ contributions | reported |
| 6a | Background | Introduction | reported |
| 7 | Objectives | Objectives | reported |
| 8 | Trial design | Study Design | reported |
| 10 | Eligibility | Methods | reported |
| 11a | Interventions | Intervention | reported |
| 12 | Outcomes | Measures | reported |
| 14 | Sample size | Pilot effectiveness Evaluation | reported |
| 16b | Allocation concealment | Study Design | reported |
| 20a | Statistical methods | Methods | reported |
| 22 | Harms | Ethics section | reported |
